# Supplementary material for: Resolving the full spectrum of human genome variation using Linked-Reads
Source: Genome Res. 2019 Apr;29(4):635–45. doi: 10.1101/gr.234443.118 (PMC6442396; doi:10.1101/gr.234443.118)
Supplement: Supplemental Material [file supp_gr.234443.118_Supplemental_longranger-2.2.zip › longranger-2.2.tar.bz2!/lr2.2-public/lib/stan/cmdstan/stan/doc/api/html/json__data__handler_8hpp_source.html]

Stan: src/stan/io/json/json\_data\_handler.hpp Source File


|  |  |
| --- | --- |
|  | Stan  2.5.0  probability, sampling & optimization |


- Main Page
- Related Pages
- Namespaces
- Classes
- Files

- File List
- File Members

All Classes Namespaces Files Functions Variables Typedefs Enumerations Enumerator Friends Macros Pages

- src
- stan
- io
- json

json\_data\_handler.hpp

Go to the documentation of this file.

1 #ifndef STAN\_\_IO\_\_JSON\_\_JSON\_DATA\_HANDLER\_HPP

2 #define STAN\_\_IO\_\_JSON\_\_JSON\_DATA\_HANDLER\_HPP

3

4 #include <cctype>

5 #include <iostream>

6 #include <limits>

7 #include <map>

8 #include <sstream>

9 #include <string>

10 #include <vector>

11 #include <boost/throw\_exception.hpp>

12 #include <boost/lexical\_cast.hpp>

13 #include <stan/math/matrix.hpp>

14 #include <stan/io/var\_context.hpp>

15 #include <stan/io/json/json\_error.hpp>

16 #include <stan/io/json/json\_parser.hpp>

17 #include <stan/io/json/json\_handler.hpp>

18

19 namespace stan {

20

21  namespace json {

22

23  namespace {

24  typedef

25  std::map<std::string,

26  std::pair<std::vector<double>,

27  std::vector<size\_t> > >

28  vars\_map\_r;

29

30  typedef

31  std::map<std::string,

32  std::pair<std::vector<int>,

33  std::vector<size\_t> > >

34  vars\_map\_i;

35

36  }

37

54  class json\_data\_handler : public stan::json::json\_handler {

55  private:

56  vars\_map\_r& vars\_r\_;

57  vars\_map\_i& vars\_i\_;

58  std::string key\_;

59  std::vector<double> values\_r\_;

60  std::vector<int> values\_i\_;

61  std::vector<size\_t> dims\_;

62  std::vector<size\_t> dims\_verify\_;

63  std::vector<bool> dims\_unknown\_;

64  size\_t dim\_idx\_;

65  size\_t dim\_last\_;

66  bool is\_int\_;

67

68  void reset() {

69  key\_.clear();

70  values\_r\_.clear();

71  values\_i\_.clear();

72  dims\_.clear();

73  dims\_verify\_.clear();

74  dims\_unknown\_.clear();

75  dim\_idx\_ = 0;

76  dim\_last\_ = 0;

77  is\_int\_ = true;

78  }

79

80  bool is\_init() {

81  return (key\_.size() == 0

82  && values\_r\_.size() == 0

83  && values\_i\_.size() == 0

84  && dims\_.size() == 0

85  && dims\_verify\_.size() == 0

86  && dims\_unknown\_.size() == 0

87  && dim\_idx\_ == 0

88  && dim\_last\_ == 0

89  && is\_int\_);

90  }

91

92

93  public:

102  json\_data\_handler(vars\_map\_r& vars\_r, vars\_map\_i& vars\_i) :

103  json\_handler(), vars\_r\_(vars\_r), vars\_i\_(vars\_i),

104  key\_(), values\_r\_(), values\_i\_(),

105  dims\_(), dims\_verify\_(), dims\_unknown\_(),

106  dim\_idx\_(), dim\_last\_(), is\_int\_() {

107  }

108

109  void start\_text() {

110  vars\_i\_.clear();

111  vars\_r\_.clear();

112  reset();

113  }

114

115  void end\_text() {

116  reset();

117  }

118

119  void start\_array() {

120  if (0 == key\_.size()) {

121  throw json\_error("expecting JSON object, found array");

122  }

123  if (dim\_idx\_ > 0 && dim\_last\_ == dim\_idx\_) {

124  std::stringstream errorMsg;

125  errorMsg << "variable: " << key\_ << ", error: non-scalar array value";

126  throw json\_error(errorMsg.str());

127  }

128  incr\_dim\_size();

129  dim\_idx\_++;

130  if (dims\_.size() < dim\_idx\_) {

131  dims\_.push\_back(0);

132  dims\_unknown\_.push\_back(true);

133  dims\_verify\_.push\_back(0);

134  } else {

135  dims\_verify\_[dim\_idx\_-1] = 0;

136  }

137  }

138

139  void end\_array() {

140  if (dims\_[dim\_idx\_-1] == 0) {

141  std::stringstream errorMsg;

142  errorMsg << "variable: " << key\_ << ", error: empty array not allowed";

143  throw json\_error(errorMsg.str());

144  }

145  if (dims\_unknown\_[dim\_idx\_-1] == true) {

146  dims\_unknown\_[dim\_idx\_-1] = false;

147  } else if (dims\_verify\_[dim\_idx\_-1] != dims\_[dim\_idx\_-1]) {

148  std::stringstream errorMsg;

149  errorMsg << "variable: " << key\_ << ", error: non-rectangular array";

150  throw json\_error(errorMsg.str());

151  }

152  if (0 == dim\_last\_

153  && ((is\_int\_ && values\_i\_.size() > 0) || (values\_r\_.size() > 0)))

154  dim\_last\_ = dim\_idx\_;

155  dim\_idx\_--;

156  }

157

158  void start\_object() {

159  if (!is\_init()) {

160  std::stringstream errorMsg;

161  errorMsg << "variable: " << key\_ << ", error: nested objects not allowed";

162  throw json\_error(errorMsg.str());

163  }

164  }

165

166  void end\_object() {

167  save\_current\_key\_value\_pair();

168  reset();

169  }

170

171  void null() {

172  std::stringstream errorMsg;

173  errorMsg << "variable: " << key\_ << ", error: null values not allowed";

174  throw json\_error(errorMsg.str());

175  }

176

177  void boolean(bool p) {

178  std::stringstream errorMsg;

179  errorMsg << "variable: " << key\_ << ", error: boolean values not allowed";

180  throw json\_error(errorMsg.str());

181  }

182

183  void string(const std::string& s) {

184  double tmp;

185  if (0 == s.compare("-inf")) {

186  tmp = -std::numeric\_limits<double>::infinity();

187  } else if (0 == s.compare("inf")) {

188  tmp = std::numeric\_limits<double>::infinity();

189  } else {

190  std::stringstream errorMsg;

191  errorMsg << "variable: " << key\_ << ", error: string values not allowed";

192  throw json\_error(errorMsg.str());

193  }

194  if (is\_int\_) {

195  for (std::vector<int>::iterator it = values\_i\_.begin(); it != values\_i\_.end(); ++it)

196  values\_r\_.push\_back(\*it);

197  }

198  is\_int\_ = false;

199  values\_r\_.push\_back(tmp);

200  incr\_dim\_size();

201  }

202

203  void key(const std::string& key) {

204  save\_current\_key\_value\_pair();

205  reset();

206  key\_ = key;

207  }

208

209  void number\_double(double x) {

210  set\_last\_dim();

211  if (is\_int\_) {

212  for (std::vector<int>::iterator it = values\_i\_.begin();

213  it != values\_i\_.end(); ++it)

214  values\_r\_.push\_back(\*it);

215  }

216  is\_int\_ = false;

217  values\_r\_.push\_back(x);

218  incr\_dim\_size();

219  }

220

221  void number\_long(long n) {

222  set\_last\_dim();

223  if (is\_int\_) {

224  values\_i\_.push\_back(n);

225  } else {

226  values\_r\_.push\_back(n);

227  }

228  incr\_dim\_size();

229  }

230

231  void number\_unsigned\_long(unsigned long n) {

232  set\_last\_dim();

233  if (is\_int\_) {

234  values\_i\_.push\_back(n);

235  } else {

236  values\_r\_.push\_back(n);

237  }

238  incr\_dim\_size();

239  }

240

241  void save\_current\_key\_value\_pair() {

242  if (0 == key\_.size()) return;

243

244  // redefinition or variables not allowed

245  if (vars\_r\_.find(key\_) != vars\_r\_.end()

246  || vars\_i\_.find(key\_) != vars\_i\_.end()) {

247  std::stringstream errorMsg;

248  errorMsg << "attempt to redefine variable: " << key\_;

249  throw json\_error(errorMsg.str());

250  }

251

252  // transpose order of array values to column-major

253  if (is\_int\_) {

254  std::pair<std::vector<int>,

255  std::vector<size\_t> > pair;

256  if (dims\_.size() > 1) {

257  std::vector<int> cm\_values\_i(values\_i\_.size());

258  to\_column\_major(cm\_values\_i,values\_i\_,dims\_);

259  pair = make\_pair(cm\_values\_i, dims\_);

260

261  } else {

262  pair = make\_pair(values\_i\_, dims\_);

263  }

264  vars\_i\_[key\_] = pair;

265  } else {

266  std::pair<std::vector<double>,

267  std::vector<size\_t> > pair;

268  if (dims\_.size() > 1) {

269  std::vector<double> cm\_values\_r(values\_r\_.size());

270  to\_column\_major(cm\_values\_r,values\_r\_,dims\_);

271  pair = make\_pair(cm\_values\_r, dims\_);

272  } else {

273  pair = make\_pair(values\_r\_, dims\_);

274  }

275  vars\_r\_[key\_] = pair;

276  }

277  }

278

279  void incr\_dim\_size() {

280  if (dim\_idx\_ > 0) {

281  if (dims\_unknown\_[dim\_idx\_-1])

282  dims\_[dim\_idx\_-1]++;

283  else

284  dims\_verify\_[dim\_idx\_-1]++;

285  }

286  }

287

288  template <typename T>

289  void to\_column\_major(std::vector<T>& cm\_vals, const std::vector<T>& rm\_vals, const std::vector<size\_t>& dims) {

290  for (size\_t i = 0; i< rm\_vals.size(); i++) {

291  size\_t idx = convert\_offset\_rtl\_2\_ltr(i,dims);

292  cm\_vals[idx] = rm\_vals[i];

293  }

294  }

295

296  void set\_last\_dim() {

297  if (dim\_last\_ > 0 && dim\_idx\_ < dim\_last\_) {

298  std::stringstream errorMsg;

299  errorMsg << "variable: " << key\_ << ", error: non-rectangular array";

300  throw json\_error(errorMsg.str());

301  }

302  dim\_last\_ = dim\_idx\_;

303  }

304

305  // convert row-major offset to column-major offset

306  size\_t convert\_offset\_rtl\_2\_ltr(size\_t rtl\_offset, const std::vector<size\_t>& dims) {

307  size\_t rtl\_dsize = 1;

308  for (size\_t i = 1; i < dims.size(); i++)

309  rtl\_dsize \*= dims[i];

310

311  // array index should be valid, but check just in case

312  if (rtl\_offset >= rtl\_dsize\*dims[0]) {

313  std::stringstream errorMsg;

314  errorMsg << "variable: " << key\_ << ", unexpected error";

315  throw json\_error(errorMsg.str());

316  }

317

318  // calculate offset by working left-to-right to get array indices

319  // for row-major offset left-most dimensions are divided out

320  // for column-major offset successive dimensions are multiplied in

321  size\_t rem = rtl\_offset;

322  size\_t ltr\_offset = 0;

323  size\_t ltr\_dsize = 1;

324  for (size\_t i = 0; i < dims.size()-1; i++) {

325  size\_t idx = rem / rtl\_dsize;

326  ltr\_offset += idx \* ltr\_dsize;

327  rem = rem - idx \* rtl\_dsize;

328  rtl\_dsize = rtl\_dsize / dims[i+1];

329  ltr\_dsize \*= dims[i];

330  }

331  ltr\_offset += rem \* ltr\_dsize; // for loop stops 1 early

332

333  return ltr\_offset;

334  }

335  };

336

337  }

338

339 }

340

341 #endif

stan::json::json\_data\_handler::end\_object

void end\_object()

Handle the end of an object.

**Definition:** json\_data\_handler.hpp:166

json\_parser.hpp

stan::json::json\_data\_handler::start\_object

void start\_object()

Handle the start of an object.

**Definition:** json\_data\_handler.hpp:158

stan::json::json\_handler

Abstract base class for JSON handlers.

**Definition:** json\_handler.hpp:15

stan::json::json\_data\_handler::set\_last\_dim

void set\_last\_dim()

**Definition:** json\_data\_handler.hpp:296

stan::json::json\_data\_handler::incr\_dim\_size

void incr\_dim\_size()

**Definition:** json\_data\_handler.hpp:279

stan::json::json\_data\_handler::key

void key(const std::string &key)

Handle the specified object key.

**Definition:** json\_data\_handler.hpp:203

stan::json::json\_data\_handler

A json\_data\_handler is an implementation of a json\_handler that restricts the allowed JSON text a set...

**Definition:** json\_data\_handler.hpp:54

stan::json::json\_data\_handler::start\_text

void start\_text()

Handle the the start of the text.

**Definition:** json\_data\_handler.hpp:109

json\_error.hpp

json\_handler.hpp

var\_context.hpp

stan::json::json\_data\_handler::null

void null()

Handle the null literal value.

**Definition:** json\_data\_handler.hpp:171

stan::math::dims

void dims(const T &x, std::vector< int > &result)

**Definition:** dims.hpp:13

stan::json::json\_data\_handler::string

void string(const std::string &s)

Handle the specified string value.

**Definition:** json\_data\_handler.hpp:183

stan::json::json\_data\_handler::boolean

void boolean(bool p)

Handle the boolean literal value of the specified polarity.

**Definition:** json\_data\_handler.hpp:177

stan::json::json\_data\_handler::save\_current\_key\_value\_pair

void save\_current\_key\_value\_pair()

**Definition:** json\_data\_handler.hpp:241

stan::json::json\_data\_handler::end\_text

void end\_text()

Handle the the end of the text.

**Definition:** json\_data\_handler.hpp:115

stan::json::json\_data\_handler::to\_column\_major

void to\_column\_major(std::vector< T > &cm\_vals, const std::vector< T > &rm\_vals, const std::vector< size\_t > &dims)

**Definition:** json\_data\_handler.hpp:289

stan::json::json\_data\_handler::convert\_offset\_rtl\_2\_ltr

size\_t convert\_offset\_rtl\_2\_ltr(size\_t rtl\_offset, const std::vector< size\_t > &dims)

**Definition:** json\_data\_handler.hpp:306

stan::json::json\_data\_handler::end\_array

void end\_array()

Handle the end of an array.

**Definition:** json\_data\_handler.hpp:139

stan::json::json\_error

Exception type for JSON errors.

**Definition:** json\_error.hpp:14

stan::json::json\_data\_handler::number\_double

void number\_double(double x)

Handle a the specified double-precision floating point value.

**Definition:** json\_data\_handler.hpp:209

stan::json::json\_data\_handler::json\_data\_handler

json\_data\_handler(vars\_map\_r &vars\_r, vars\_map\_i &vars\_i)

Construct a json\_data\_handler object.

**Definition:** json\_data\_handler.hpp:102

stan::json::json\_data\_handler::start\_array

void start\_array()

Handle the start of an array.

**Definition:** json\_data\_handler.hpp:119

stan::json::json\_data\_handler::number\_long

void number\_long(long n)

Handle a the specified long integer value.

**Definition:** json\_data\_handler.hpp:221

stan::json::json\_data\_handler::number\_unsigned\_long

void number\_unsigned\_long(unsigned long n)

Handle a the specified unsigned long integer value.

**Definition:** json\_data\_handler.hpp:231

matrix.hpp


---

[ Stan Home Page ]

*© 2011–2014,
Stan Development Team.*
